# Superficial Temporal Artery to Middle Cerebral Artery (STA-MCA) bypass: How I do it

**DOI:** 10.1007/s00701-026-06799-2

**Published:** 2026-02-17

**Authors:** Paolo Palmisciano, Bruno Vernile, Sudhakar Vadivelu, Mario Zuccarello

**Affiliations:** 1 Department of Neurosurgery, University of California Davis, Sacramento, CA USA; 2 Department of Neurosciences, Biomedicine and Movement Sciences, Institute of Neurosurgery, University of Verona, Verona, Italy; 3 Department of Pediatric Neurosurgery, Cincinnati Children’s Hospital Medical Center, 3333 Burnet Ave, Cincinnati, OH 45229 USA

**Keywords:** Atherosclerosis, Bypass, Microsurgery, Moya-Moya disease, Stroke

## Abstract

The superficial temporal artery–middle cerebral artery (STA–MCA) bypass remains a key extracranial–intracranial revascularization technique for moyamoya disease, complex aneurysms, and symptomatic steno-occlusive pathology not amenable to endovascular treatment. Effective bypass requires detailed knowledge of STA and MCA anatomy, comprehensive preoperative imaging, and meticulous microsurgical planning. We describe a stepwise operative strategy including donor and recipient vessel preparation, microanastomosis, and intraoperative patency assessment. Technical nuances, complication avoidance, and postoperative management are emphasized. This report outlines essential principles for safe and durable STA–MCA bypass revascularization.

**Supplementary Information:**

The online version contains supplementary material available at 10.1007/s00701-026-06799-2.

## Introduction

The superficial temporal artery to middle cerebral artery (STA–MCA) bypass is a foundational extracranial–intracranial (EC–IC) revascularization technique [8], originally described by Yasargil and Donaghy [7] to augment cerebral perfusion in ischemic disease and to provide collateral flow when parent vessel sacrifice is required. Despite its technical demands, it remains essential in the management of moyamoya disease, complex aneurysms not amenable to endovascular therapy, and symptomatic steno-occlusive disease unresponsive to medical treatment. The STA–MCA bypass has represented a model for the refinement of microsurgical principles, requiring delicate vessel handling, precise suture placement, and meticulous hemostasis. The procedure remains an essential skill for vascular neurosurgeons, representing the enduring relevance of open revascularization in an era increasingly dominated by endovascular interventions. We describe our institutional technique to perform a STA-MCA bypass.

## Surgical anatomy

A comprehensive understanding of both donor and recipient vessels, with their surrounding anatomic relationships, is essential for performing a safe and effective extracranial–intracranial (EC–IC) bypass.

The superficial temporal artery (STA), a terminal branch of the external carotid artery, courses anterior to the external auditory canal and ascends superior to the zygomatic arch. It typically bifurcates into frontal and parietal divisions approximately 1–3 cm above the zygoma, usually selected as the donor vessel. Owing to its superficial position within the subcutaneous tissues, the STA is readily accessible; however, meticulous dissection is required to prevent vasospasm, preserve adequate vessel length, and maintain luminal integrity for the anastomosis. During exposure, meticulous interfascial or subfascial dissection to avoid injuring the frontal branch of the facial nerve, which traverses the zygomatic arch near the STA [10]. The STA overlies the temporalis fascia and muscle, which must be carefully mobilized to create a tension-free graft while preserving temporalis function and cosmetic appearance.

The middle cerebral artery (MCA) serves as the recipient artery. Originating from the internal carotid artery and coursing laterally through the sylvian fissure, its M4 cortical branches on the lateral convexity are typically used for STA–MCA bypass [9]. These branches lie adjacent to cortical veins and are enveloped by delicate pia–arachnoid membranes, necessitating precise, atraumatic microsurgical dissection under high magnification. An optimal recipient is one with sufficient caliber, favorable flow characteristics, and a location that permits a short, tension-free graft orientation.

The bony architecture of the temporal squama and pterional region defines the operative corridor. A craniotomy tailored to the STA trajectory and the underlying MCA cortical territory affords the most direct and efficient exposure for the anastomosis.

## Surgical technique

### Preoperative assessment

A comprehensive neurological examination is essential to establish a baseline and to identify deficits that may influence perioperative management. Optimization of medical comorbidities should be completed preoperatively.

#### Preoperative vascular imaging:

- CTA and Digital Subtraction Angiography (DSA) are performed to characterize donor and recipient vessels, including STA anatomy, branching pattern, bifurcation morphology, branch length, and luminal diameter. DSA is important for assessing transdural collateral networks, which must be preserved during dural opening.
- Preoperative vascular mapping informs incision planning based on the scalp trajectory of the STA.

#### Cerebral perfusion assessment:

**Fig. 1:**
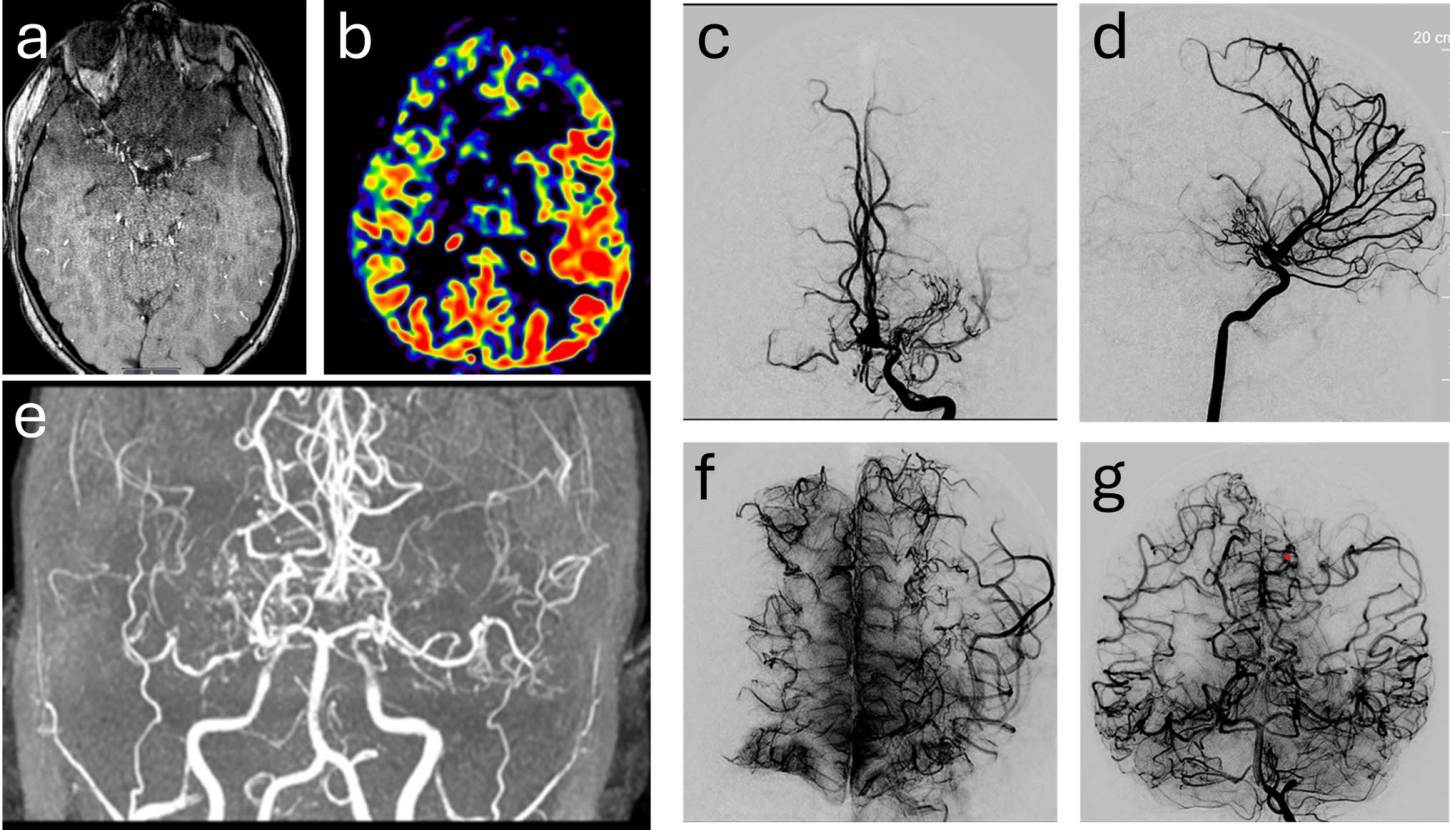
Preoperative assessment of cerebral perfusion and recipient MCA matching for donor vessel length measurement. MRA evaluation of bilateral ICA steno-occlusive disease with abundant moyamoya collaterals (***a***), ASL left hemispheric cerebral perfusion asymmetry (***b***), and DSA cerebral angiography of delayed hypoperfusion in the left cerebral hemisphere (***c*** and ***d***). MRA coronal view demonstrating paucity of adequate MCA recipient vessels (***e***), however DSA cerebral angiography demonstrating adequate MCA recipients on coronal views in the ICA and Vertebral artery injections (***f*** and ***g***)

- CT perfusion, MR perfusion, or SPECT are used to evaluate regional cerebral blood flow, cerebrovascular reactivity, and the severity of hemodynamic impairment.
- MRI is obtained to evaluate parenchymal integrity and exclude recent ischemia. Coronal imaging is useful in longstanding moyamoya disease or cerebral atrophy to estimate the usable length of the donor STA branch and assess potential recipient sites without significant infarction (Fig. 1).

### Pre-procedure plan

1. The patient is placed supine with the head elevated 10–20° and rotated approximately 90° so that the operative field is parallel to the floor.
2. Intraoperative monitoring.
3. Normotension and normocapnia are maintained to preserve cerebral perfusion.

### Our technique

#### Donor Vessel (STA) harvest

Preoperative Doppler ultrasonography is used to map the STA and identify the optimal branch. After anesthesia induction, a skin incision is made along the mapped course. The donor branch is dissected meticulously, with preservation of adventitia and control of side branches. The STA is kept continuously moist to minimize vasospasm.

#### Craniotomy

A tailored craniotomy is fashioned over the anticipated cortical MCA branch. After lifting the bone flap, the dura is opened with preservation of dural vascular channels when possible.

#### Recipient Vessel (MCA) exposure

MCA cortical branches are identified based on accessibility, diameter (≥ 1 mm preferred), and proximity to the STA. Arachnoid dissection is performed under high magnification to mobilize the vessel without injuring adjacent cortical veins. Pre-bypass evaluation with indocyanine green (ICG) angiography may assist in assessing local collateral flow and selecting the optimal recipient.

#### Donor and recipient vessels preparation

**Fig. 2:**
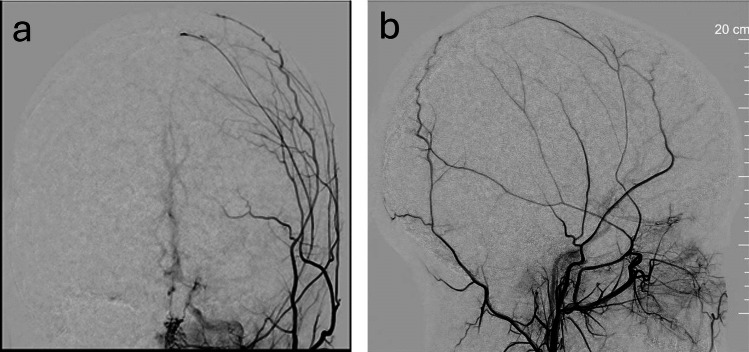
Frontal (***a***) and lateral (***b***) DSA cerebral angiographic evaluations of the donor parietal STA branch that appears small but adequate

**Fig. 3:**
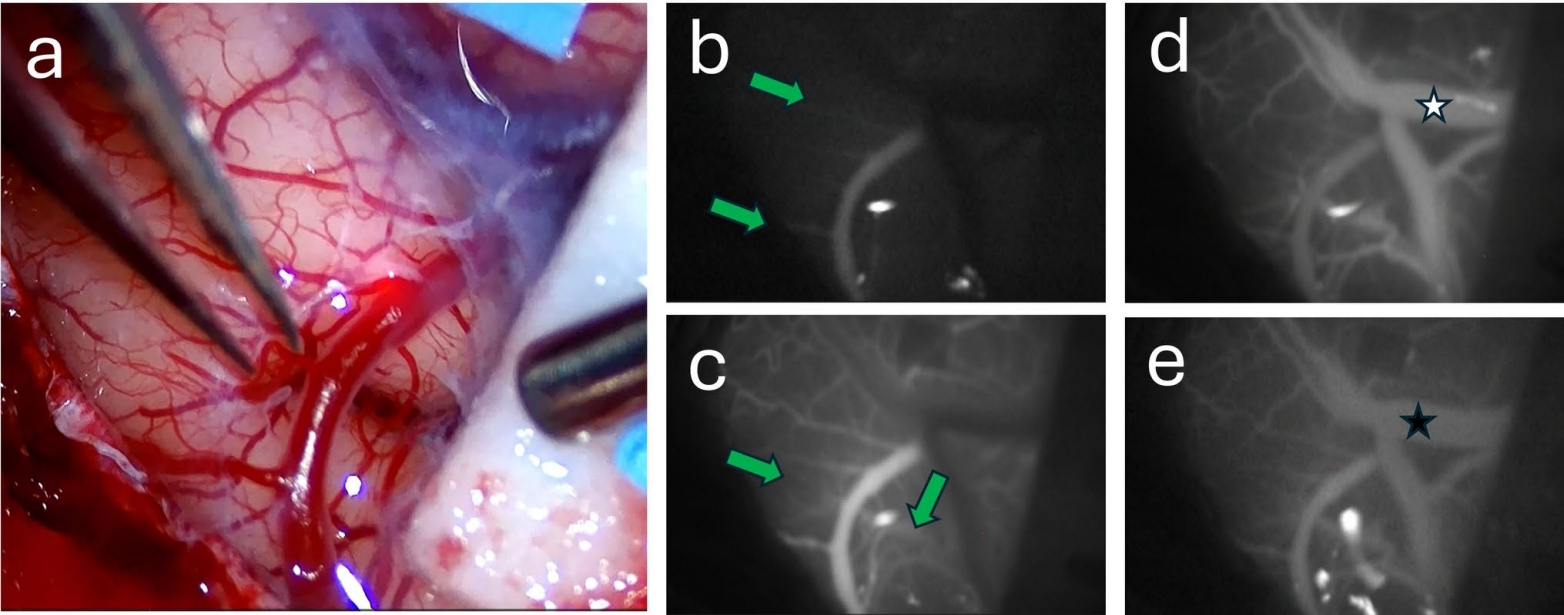
Intraoperative MCA recipient assessment. Please note the hyperemic appearance of the cerebral cortex in a moyamoya patient with multiple MCA arterial cortical tributaries (***a***). ICG video analysis of the early arterial phase (***b***) demonstrating dominant cortical arterial tributaries versus late arterial phase with now additional less dominant cortical arterial tributaries (***c***); green arrows demarcate those cortical tributaries. Venous phase ICG video analysis with appearance of venous outflow (***d***), please note the white star overlying the bright opacification of the vein while over time the same vein with a black star appears less intense in opacification to the original MCA arterial phase (***e***). This MCA arterial opacification persistence demonstrates the abnormal cerebral cortical reserve capacity in moyamoya disease

The STA is temporarily occluded proximally to prevent backflow and reduce thrombogenicity. It is irrigated with heparinized saline. If the donor diameter is < 1 mm, the end is trimmed at a 45° angle to enlarge the anastomotic surface area. Use of topical papaverine and mechanical intraluminal dilation may be performed to aid in small donor STA preparation (Fig. 2). Recipient vessels are evaluated for size, and small collateral cortical arteries can be evaluated with ICG (Fig. 3). The MCA branch is cross-clamped and arteriotomy is performed using a 30-gauge needle. The luminal edge may be stained lightly with methylene blue to aid visualization.

#### Microanastomosis

An end-to-side anastomosis is constructed using 10–0 nylon under the operating microscope. Two opposing stay sutures are placed first, followed by additional interrupted stitches (typically 6–8) to achieve a watertight seal [4].

#### Patency assessment

**Fig. 4:**
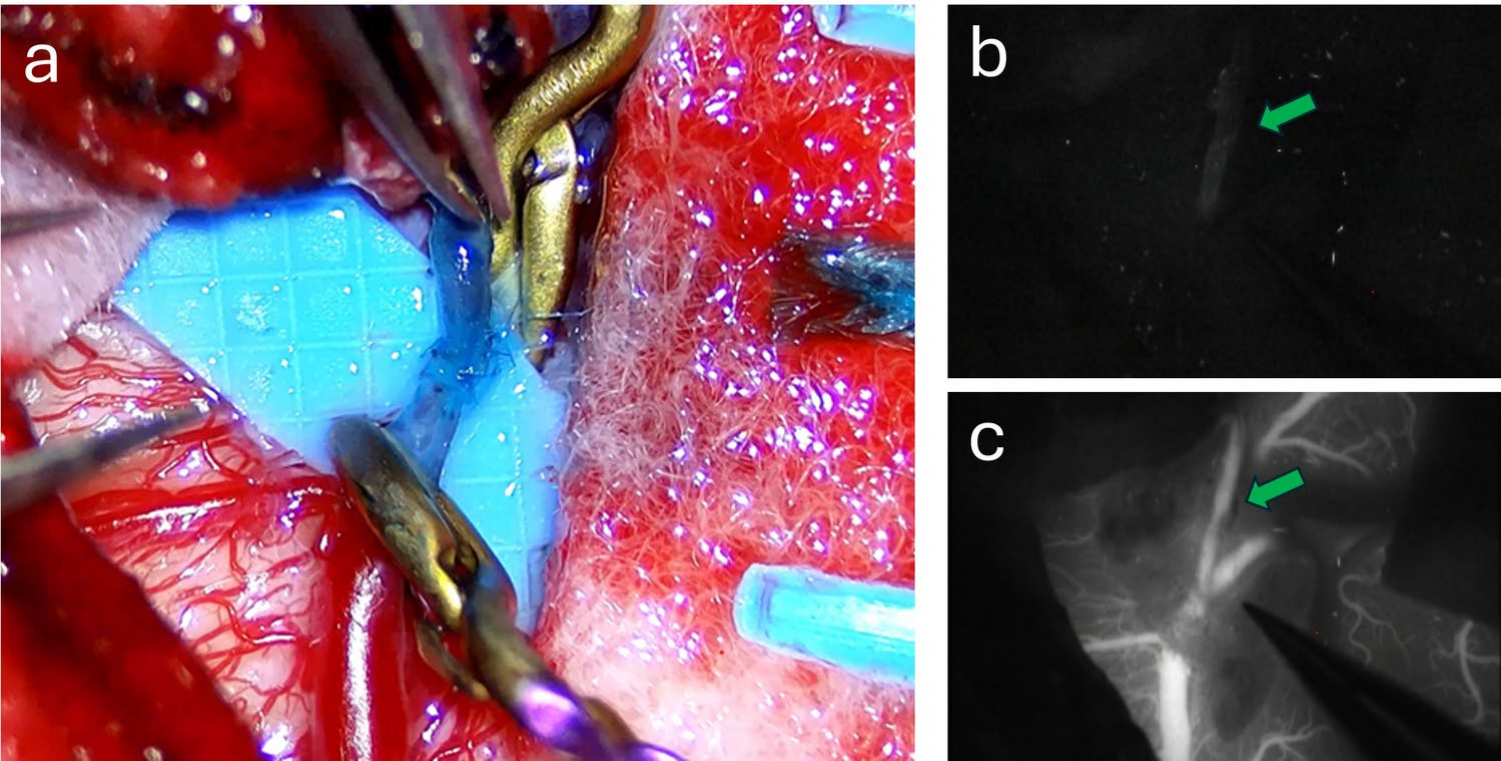
Newly constructed STA – MCA direct end to side anastomosis (***a***). ICG video analysis demonstrating initial opacification of the donor STA (***b***) green arrow compared to subsequent opacification of the full STA – MCA cerebral revascularization bypass (***c***)

Bypass function is verified intraoperatively using micro-Doppler ultrasonography and ICG angiography. ICG of the constructed bypass can be assessed for Type I cerebral revascularization bypass flow, noting anterograde flow from the STA to the MCA without impedance (Fig. 4) [3, 5]. After confirming flow, the bone flap is replaced with a tapered channel or bypass plate to prevent kinking of the STA. The dural closure is intentionally lax to avoid compression. The STA course is re-examined during closure to ensure the graft is neither redundant nor tensioned.

#### Wound closure

Once the anastomosis is complete and patency is confirmed, the dura mater is partially closed, the bone flap is replaced, and the scalp is sutured. Special attention is given to avoid any kink of the STA during the closure.

#### Early postoperative management

Focus on maintaining adequate cerebral perfusion while preventing hyper-perfusion and graft thrombosis. The patient is admitted in ICU for strict blood pressure control, hourly neurological examinations, normocapnia, and euvolemia. Early CTA is obtained to assess graft patency. Antiplatelet therapy is continued unless contraindicated by hemorrhagic complications.

### Indications

- Symptomatic hemodynamic impairment due to vessel occlusion or stenosis when endovascular treatment is not feasible [8].
- Moyamoya disease [2].
- Complex cerebral aneurysms not amenable to endovascular repair [1].
- Select skull base tumors requiring flow replacement or vascular augmentation [6].

### Limitations

- Graft occlusion, which may compromise the revascularized territory
- Unsuitable donor or recipient vessels (i.e., branch diameters < 1 mm)
- Prior surgeries or scarring that limit STA length or safe exposure

### How to avoid complications

- Maintain normotension and normocapnia throughout the procedure to optimize cerebral perfusion.
- Preoperatively start antiplatelet therapy (e.g., aspirin) unless contraindicated to prevent blood clots.
- Use meticulous atraumatic handling of the STA and MCA branches and precise microsuturing techniques.
- Assess bypass patency intraoperatively and during wound closure; inspect for leakage that might predispose to subdural collections.
- Schedule regular follow-up neurovascular imaging to check bypass graft patency.
- Perform careful soft-tissue closure to avoid wound ischemia or breakdown.
- Maintain stable postoperative blood pressure to prevent hemorrhagic complications

### Patient information

Patients should be informed of potential risks and complications:

- Ischemic or hemorrhagic complications causing paralysis, speech impairment, cognitive dysfunction
- Hyperperfusion syndrome
- Seizures
- Cerebrospinal fluid leakage
- Skin and wound-healing complications
- Possibility of re-operation

### Key Points

1. Appropriate patient selection based on hemodynamic impairment
2. Pre-operative antiplatelets therapy
3. Strict intra-operative normocapnia and normotension
4. Meticulous micro-surgical technique
5. Intra-operative verification of bypass patency
6. Post-operative neurological examination

## Supplementary Information

Below is the link to the electronic supplementary material.

Supplementary file1 Video 1. Description of a right-sided approach with STA-MCA anastomosis for treatment of patient with Moya-Moya disease. (MP4 259630 KB)

## Data Availability

Data materials are available per direct requests to the authors.

## Abbreviations

CTA: Computer tomography angiography
DSA: Digital subtraction angiography
EC–IC: Extracranial–intracranial
ICG: Indocyanine green
MCA: Middle cerebral artery
STA: Superficial temporal artery

## References

[1] Andaluz N, Zuccarello M (2011) Treatment strategies for complex intracranial aneurysms: review of a 12-year experience at the University of Cincinnati. Skull Base 21(04):233–242. 10.1055/s-0031-1280685 22470266 10.1055/s-0031-1280685 PMC3312119

[2] Andaluz N, Choutka O, Zuccarello M (2010) Trends in the management of adult Moyamoya disease in the United States: results of a nationwide survey. World Neurosurg 73(4):361–364. 10.1016/j.wneu.2010.01.005 20849794 10.1016/j.wneu.2010.01.005

[3] Cavallo C, Gandhi S, Zhao X et al (2019) Applications of microscope-integrated indocyanine green videoangiography in cerebral revascularization procedures. Front Surg. 10.3389/fsurg.2019.00059 31850362 10.3389/fsurg.2019.00059 PMC6902023

[4] Gross BA, Du R (2012) STA-MCA bypass. Acta Neurochir (Wien) 154(8):1463–1467. 10.1007/s00701-012-1412-3 22688612 10.1007/s00701-012-1412-3

[5] Januszewski J, Beecher JS, Chalif DJ, Dehdashti AR (2014) Flow-based evaluation of cerebral revascularization using near-infrared indocyanine green videoangiography. Neurosurg Focus 36(2):E14. 10.3171/2013.12.FOCUS13473 24484252 10.3171/2013.12.FOCUS13473

[6] Moritake K, Handa H, Yamashita J et al (1984) STA-MCA anastomosis in patients with skull base tumours involving the internal carotid artery? Haemodynamic assessment by ultrasonic Doppler flowmeter. Acta Neurochir (Wien) 72(1–2):95–110. 10.1007/BF01406817 6741650 10.1007/BF01406817

[7] Peardon Donaghy RM, Yasargil G. Microangeional surgery and its techniques. In: 1968:263–267. 10.1016/S0079-6123(08)61469-7 10.1016/s0079-6123(08)61469-7 5735457

[8] Powers WJ, Clarke WR, Grubb RL et al (2011) Extracranial-intracranial bypass surgery for stroke prevention in hemodynamic cerebral ischemia. JAMA 306(18):1983. 10.1001/jama.2011.1610 22068990 10.1001/jama.2011.1610 PMC3601825

[9] Rubio RR, Lawton MT, Kola O et al (2018) The middle temporal artery: surgical anatomy and exposure for cerebral revascularization. World Neurosurg 110:e79–e83. 10.1016/j.wneu.2017.10.100 29111474 10.1016/j.wneu.2017.10.100

[10] Shin K, Shin HJ, Lee S, Koh K, Song W (2018) Surgical anatomy of the superficial temporal artery to prevent facial nerve injury during arterial biopsy. Clin Anat 31(4):608–613. 10.1002/ca.23033 29226469 10.1002/ca.23033

